# Reporting Characteristics in Sports Nutrition

**DOI:** 10.3390/sports6040139

**Published:** 2018-11-05

**Authors:** Conrad P. Earnest, Brandon M. Roberts, Christopher R. Harnish, Jessica L. Kutz, Jason M. Cholewa, Neil M. Johannsen

**Affiliations:** 1 Exercise and Sports Nutrition Laboratory, Texas A&M University, College Station, TX 77843, USA; 2 UAB Center for Exercise Medicine, University of Alabama at Birmingham, Birmingham, AL 35294, USA; brob21@uab.edu; 3 Department of Exercise Science, Shenandoah University, Winchester, VA 22601, USA; charnish@su.edu (C.R.H.); jkutz@su.edu (J.L.K.); 4 Department of Kinesiology, Recreation, and Sport Studies, Coastal Carolina University, Conway, SC 29528, USA; jcholewa@coastal.edu; 5 School of Kinesiology, Louisiana State University, Baton Rouge, LA 70803, USA; njohan1@lsu.edu

**Keywords:** nutrition, methodology, performance, statistics

## Abstract

**Purpose**: To examine data reporting characteristics in sports nutrition. **Methods**: We examined 236 papers from ten journals published in 2016. The primary outcome was statistical variance associated with treatment (SD (correct) vs. SEM or CI). Secondary outcomes included the reporting of: (a) effect sizes (Y/N); (b) outcome prioritization (Y/N; primary, secondary, etc.) and (c) statistical variance relative to change from baseline (CI (correct) vs. SD or SEM). As tertiary/exploratory outcome, we examined whether authors stated a directed hypothesis. Statistical evaluation was performed using chi-square analyses. **Results**: We observed significant trends for all analyses (*p* < 0.001) and between category comparisons (*p* < 0.002). For the primary outcome, 128 (59%) articles correctly used SD to denote treatment variance, while 79 (36%) and 11 (5%) used SEM and CI, respectively. For secondary outcomes, 63 articles (29%) reported effect sizes, while 155 (71%) did not. Additionally, 188 articles (86%) did not prioritize outcomes, 134 articles (61%) stated no hypotheses and 40 (19%, out of 100) articles used CI to denote change scores vs. SD (19%, *n* = 41) and SEM (*n* = 10, 5%). Eight articles (4%) reported no variance terms. **Conclusions**: Overall, there are gaps regarding reporting in sports nutrition. Editors, journal publishers, and the field of exercise science alike should consider these outcomes and provide editorial staff, reviewers and authors with more concrete guidelines.

## 1. Introduction

The advocacy for consistent reporting is not new to science. In 1983, Altman and colleagues published two papers suggesting that the medical sciences would benefit from more consistent data reporting [1,2]. Subsequently, the promotion of better data reporting has materialized in medical journals [1], but a paucity of papers promote similar stringency in sports nutrition. This is important as Gore et al., in a paper to the Lancet (1992), recognized that the reported methods and results were inadequate in about half of the papers making major errors of inference [3]. The same authors further observed that the descriptions of methods and results were found to be inadequate in ~50% of papers, and ~25% of papers demonstrated inadequate abstracts and conclusions. Why is this important?

Interpreting research studies involving a treatment is dependent on trial design and the accurate reporting of study findings. These factors are important to exercise scientists, practitioners (i.e., coaches, nutritionists, etc.) and ultimately any athlete looking for reliable information when making decisions regarding dietary strategies targeting improved sports performance. An essential component to interpreting the research findings of any study is their accurate presentation. On an intuitive level, consistency across journals relative to reporting standards should be the norm; however, we contend there is a lack of consistency regarding data reporting in sports nutrition trials using experimental designs aimed at improving performance.

The aim of our study was to examine the reporting characteristics of sports nutrition reports employing a treatment targeting sports performance. Accordingly, we examined several key factors that in our opinion would have an immediate impact on the reporting characteristics of sports nutrition trials. Our primary outcome was the reporting of treatment variance via standard deviations (SD) vs. standard error of the mean (SEM) or confidence intervals (CI) as the latter two terms are inferential statistics and do not adequately address the variance of a treatment condition [4,5]. We also examined several secondary outcomes including the reporting of: (1) effect sizes, (2) structured/prioritized outcomes via the identification of primary and secondary outcomes, (3) direct hypotheses and (4) variance components relative to the reporting of change scores. We hypothesized that a significant number of articles use SEM and CI instead of SD, and do not report effect sizes, structured analyses, directed hypotheses, or correct variance terms surrounding changes scores whether expressed as mean or percentage.

## 2. Methods

**Figure 1:**
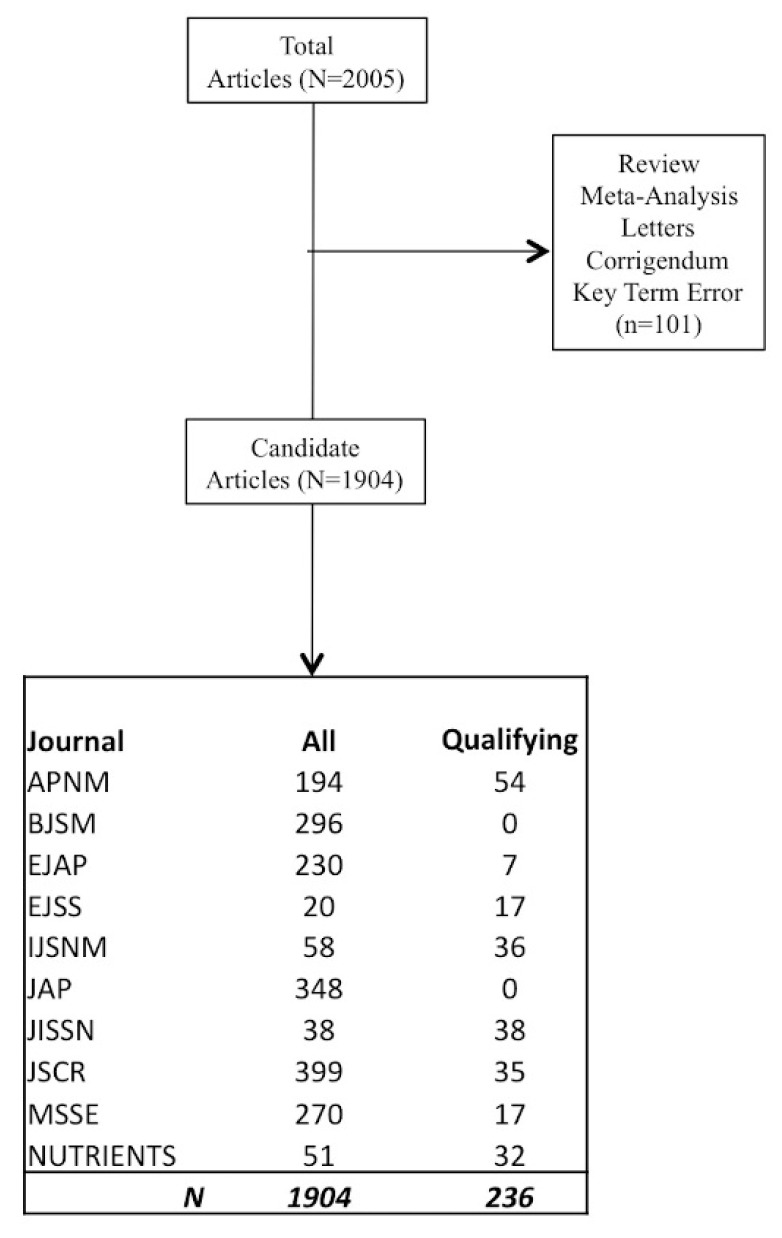
Schematic presentation analyzed journals articles. Journals are Applied Physiology, Nutrition and Metabolism (APNM), British Journal of Sports Medicine (BJSM), European Journal of Applied Physiology (EJAP), European Journal of Sports Science (EJSS), International Journal of Sports Nutrition and Exercise Metabolism (IJSNM), Journal of Applied Physiology (JAP), Journal of the International Society of Sports Nutrition (JISSN), Journal of Strength and Conditioning Research (JSCR), Medicine Science Sports and Exercise (MSSE), and Nutrients.

To perform our analysis, we examined articles from the year 2016 in order to examine a full year of reporting. Data collection was initiated in September of 2017 and concluded in March 2018. Initially, we ranked journals according to impact factor. Depending on the number of articles published within each journal, we further extended our search to include other journals demonstrating a propensity for publishing sports nutrition reports. We have presented a flow chart of the examined journals in Figure 1. All of these journals were available on PubMed and a full copy of each article was inspected for our proposed outcomes. Data was initially queried using the following key words: Diet, dietary, nutrition, supplement, carbohydrate, protein, placebo, fat, hydration, human, and clinical trial providing us with 2005 candidate articles. Subsequently, the candidate articles were then queried for terms such as review, meta-analysis, letter to the editor, etc. and eliminated if present. This provided us with 1904 potential articles. Finally, the remaining articles were examined one-by-one until the representative pool of 236 articles was established.

### 2.1. Primary Outcome

The primary outcome for our analysis was the reporting of error terms to denote treatment/measurement variance; specifically, the use of three typically used terms: (1) standard deviations (SD) vs. (2) standard error of the mean (SEM) or (3) confidence intervals (CI). The SD is a descriptive statistic describing the spread of a distribution surrounding the treatment. The SEM and CI are inferential statistics when comparing sample means across populations [4,5]. In essence, the SD describes the variability between individuals in a sample and the SEM describes the uncertainty of how the sample mean represents the population mean [6]. As a consequence, the SEM is always less than the SD, and it misleads readers to underestimate the variability between individuals within the study sample relative to a treatment/intervention.

### 2.2. Secondary Outcomes

Secondary analyses were then performed on the following four factors.

(1) Did the authors report an effect size for their analysis? This was categorized as Yes or No.
(2) Did the authors stratify or prioritize their analysis as a primary (i.e., most important), secondary, tertiary (exploratory), etc. analyses? This was categorized as Yes or No.
(3) If reporting change scores (mean or percentage), did the authors use confidence intervals or did they report SD or SEM.
(4) Did the authors state a directed, a priori hypothesis for their paper? Observations were categorized as: (a) directed hypotheses; (b) non-specific/general hypothesis or (c) no hypothesis stated.

A directed hypothesis was defined as one having a direct cause and specific effect relationship relative to the identified outcomes of the study. For example: “We hypothesized that the ingestion of carbohydrate during a time trial would improve cycling time to completion of the trial?” Hypotheses were considered non-specific or general if the authors did not identify a specific outcome. For example, “We hypothesized that the ingestion of carbohydrates during a 60 km time trial would improve time trial performance” and then examine a number of non-prioritized factors such as time to complete, average power output, pedal cadence, etc. Finally, many papers use “aims” as a synonymous term to hypothesis. Though this is not technically correct (see Section 5, Discussion), we applied the aforementioned criteria to those papers stating an aim.

## 3. Statistics

We performed a chi-square for the prevalence of responses with *p* < 0.05 representing statistical significance. For questions involving more than two categories, we performed pairwise category comparisons using chi-square analyses with Bonferonni corrections for multiple comparisons. For example, the primary outcome and our question regarding hypotheses were each composed of three possible responses (see Introduction). Prevalence is denoted as the occurrence of each finding as a percentage of the total possible responses. For these latter two analyses, the chi-square denotes an overall p-for-trend, with between category pairwise comparisons requiring individual chi-square assessments. Hence, we adjusted these two analyses using Bonferonni corrections and established significance at <0.017. Finally, we considered a journal-by-journal comparison. This was not possible for several questions as there were an insufficient number of responses in some cells to adequately represent a clear comparison and would unfavorably bias such an analysis. We think the global reporting characteristics are more important to the field than comparing the journals to one another.

## 4. Results

**Figure 2:**
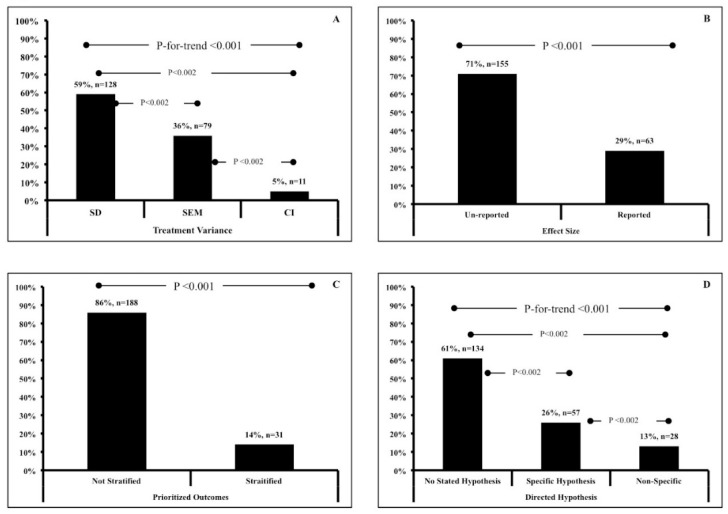
Data represent frequency (%, *n*) of (**A**) reported treatment variance; (**B**) reported effect size, (**C**) prioritized outcome stratification and (**D**) reported hypotheses.

We have presented our findings in Table 1. Overall, we observed a significant trend for all of the analyses (*p* < 0.001; Figure 2). For our primary outcome, 128 (59%) articles used SD to denote treatment variance, while 79 (36%) and 11 (5%) articles used SEM and CI, respectively (Figure 2A). For our secondary outcomes, 63 articles (29%) reported effect sizes while 155 (71%) did not (Figure 2B), Similarly, 188 articles (86%) did not prioritize outcomes (Figure 2C), 134 articles (61%) stated no hypotheses (Figure 2D), and 40 (19%, out of 100) used CI to denote change from baseline, while 41 (19%) used SD and 10 (5%) used SEM. Eight articles (4%) reported no variance terms at all. For the primary outcomes and the question regarding hypotheses, all between category comparisons were significant (*p* < 0.002).

## 5. Discussion

Our goal was to examine a number of data reporting characteristics relative to intervention trials involving sports nutrition. Our results demonstrate that a significant number of papers use SEM and CI incorrectly as a measure of treatment variance, do not report effect sizes or use stratified analyses, and inadequately denote data examining change. Furthermore, a large percentage of reports (61%) did not state a hypothesis at all or did so non-specifically (13%). Therefore, we accept our research hypothesis that a significant number of journal articles do not engage in robust reporting guidelines and demonstrate a number of inconsistencies across journals reporting on sports nutrition. These findings are important and we propose that adopting better reporting standards will benefit the sports nutrition sciences and exercise sciences as a whole.

### 5.1. Primary Outcome

The advancement and visibility of sports nutrition has grown continually since the early 1960’s largely due to better analytical methods. Examples include, the refinement of intra and extra-muscular techniques to measure various analytes has evolved as well as a continued interest in genetics. It is our opinion that reporting standards should not be neglected either. Starting with our primary outcomes, it is evident that the hierarchy of data reporting is either not adhered or understood well. For example, SEM is not a descriptive statistic but is an inferential statistic [4,7]. Even then, confidence intervals are recommended instead of SEM [6]. Some suggest that the SEM should be reported so that a confidence interval can be calculated [8]. While this is technically correct, we contend that from a practical standpoint, reporting the actual SD and CI decreases the reader’s burden of having to calculate it for from the SEM and improves the available information for those wishing to perform meta-analyses. Anecdotally, we have also observed that a common strategy is to report SD in tables; yet, SEM in the graphics. This may mislead readers into the illusion that the variance surrounding the treatment of an intervention is smaller than the true variance of the treatment.

The most important aspect of this discussion is that of all the variance terms involved in any intervention study, the SD is the correct statistical reporting measure since it is a measure of treatment variability [1,4,9,10,11,12]. Importantly, the misuse of reporting SEM vs. SD is not new to science. In the field of anesthesia, for example, Nagele reported that, ~23% of published articles, across four journals, used SEM incorrectly instead of SD [6]. This is lower than the 36% we observed in our current report. Finally, we would like to note that the CONSORT Statement also recommends reporting the SD for studies utilizing parallel group randomized trials since these type of trials are often used in sports nutrition [13].

### 5.2. Secondary Outcomes

#### 5.2.1. Effect Sizes

The inclusion of effect sizes are also advocated by the CONSORT Statement, as well as others groups [4,13]. However, from our analysis, sports nutrition intervention reporting has not kept pace with such advocacy. Common effect sizes in intervention studies are Cohen’s d (t-tests with equal sample variance), Hedge’s g (small sample t-tests with unequal sample sizes), partial eta squared (n-way ANOVA), and odds ratio’s for studies using logistic regression [14]. This, of course, does not preclude the use of Pearson’s r (linear correlation) and Spearman’s rho (rank correlation). Intervention trials are often designed to examine the before-and-after (i.e., pre/post) effects of a treatment. While effect sizes are not exclusive to intervention studies, the inclusion of such findings is important to sports nutrition studies and trials involving smaller sample sizes should not discount spurious findings if data is interpreted solely on *p*-values [15]. While it is not our intent to dissuade readers from using *p*-values, it is our intention to suggest that the inclusion of *p*-values and effect sizes serve as a compliment to ones research findings. For further reading on this topic, we recommend the American Statistical Associations 2016 Statement [16].

#### 5.2.2. Hypotheses and Stratified Analyses

**Figure 3:**
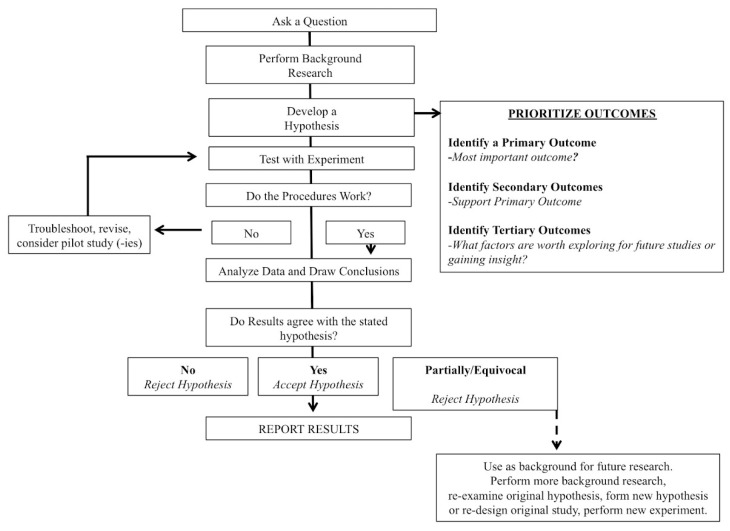
Schematic representation of the scientific method.

Why are hypotheses and stratified/prioritized analyses important? A common tenet of all scientific disciplines is (or should be) the scientific method (Figure 3). While some may feel that this assertion is pedantic, it is our opinion that the scientific method should be a cornerstone of sports nutrition and the exercise sciences; yet, we are also of the opinion that the scientific method is often overlooked or inadequately reported. As proposed by Darwin, the scientific method first consists of formulating, followed by the subsequent experimentation to test the hypothesis by empirical testing [17]. A central principle of the scientific methods is the collection of empirical data to test a hypothesis. Or, as put forth by Supino (2012), the hypothesis is a construct, interposed between a problem and its solution, which represents a proposed answer to a specific research question [18]. Farrugia (2010) further suggests that the research question should be developed before the start of the study and the primary objective of the study should be coupled (i.e., directed) to the hypothesis in order to communicate the clinical relevance of the trial [19]. Clark (2011) echoes these sentiments and goes further to state that very few papers formally reject or accept their hypothesis in the discussion, which, when done, shows a true understanding of research [20]. Anecdotally, within our roles as reviewers and editors, we have observed that it is not uncommon to formulate hypotheses after submission and review. This *ex post facto* practice is clearly not a valid method and should be avoided. We also observed that many papers stated an “aim,” but the aim was usually non-specific. What’s wrong with an aim? 

Strictly defined, an aim is a clearly directed intent or purpose and, in some cases, could be used synonymously with hypothesis under the proviso that the aim is specific and directed. However, the absence of a specific aim, coupled to outcome prioritization, and disconnected from a specific, research driven hypothesis, lends itself to a form of “shotgun science,” whereby investigators examine a myriad of outcomes and choose the one they like best. In order to alleviate such potential misunderstandings, the aforementioned triad of characteristics would eliminate such doubt. We contend that ‘the hypothesis is not dead’ and should remain a focal point of scientific reporting.

Despite these simple principles, we observed that a significant number of papers (61%) stated no hypothesis whatsoever, and 13% of those papers stating a hypothesis, did in a non-specific manner. In the sports sciences, a well-formed hypothesis should be a natural extension of a specific research question. In turn, this brings clarity to study design, facilitates the reading of a paper, and helps the researchers themselves understand what the results of a study indicate. Moreover, a clear hypothesis can help mitigate inherent biases by setting an obvious threshold for the research to reach. Based on our analysis, well over half of the papers surveyed either do not state, or at best, provided a poorly stated ambiguous hypothesis versus one that is clear and guided. As most sports nutrition studies utilize some type of intervention, one should expect an evidence-based hypothesis to be a distinct benchmark in order to reject null hypothesis. We do not wish to imply that the lack of a hypothesis or prioritized approach is conspiratorial, but rather simply unfocused and prone to study misinterpretation. Ultimately, this would assist readers in interpreting any published paper as a focused hypothesis and structured prioritization of outcomes work hand-in-hand.

A typical feature of sports nutrition research is to examine multiple outcomes related to a treatment. Such outcomes may be related to indices of performance, as well as bio-mechanistic measures targeting potential mechanisms of action. Establishing a prioritized analysis schema demonstrates several cogent factors. First, it communicates to the reader that the investigators have thought through what is the most important aspect of the study and other factors that might be supportive of such findings. Second, it strengthens *a priori* analyses for future studies. Lastly, it attenuates potential criticism if any of the studied parameters are exploratory in nature (i.e., tertiary analyses), which may not be adequately powered within a study, but may hold promise for future studies. Hence, establishing a hierarchy should be determined during the design of the study [21]. In our current analysis, we found that 86% of the articles examined did not demonstrate any prioritized outcomes, which is also recommended within the CONSORT Statement, and it is further suggested that researchers use a single primary outcome to avoid interpretation association with multiplicity of analyses and acknowledgement of additional secondary outcomes [13]. This strategy has several additional strengths.

By setting the primary outcome in advance, this limits the opportunity for investigators to choose the significant results that they like following the completion of a study. Subsequently, a prioritization approach decreases potentially spurious reports that may be due to “cherry picking,” which is a form of “p-hacking” despite the intentions of the researchers performing the study [21]. In addition, there needs to be transparency in how power calculations are determined, which should be based on the primary outcome specified in the design of the study. If a power analysis is conducted for a primary outcome but a secondary outcome is chosen to be reported for statistical significance, this may yield false-negative results [22]. Studies that are exploratory in nature require specific scrutiny for the risk of false-negative or false-positive results [22]. The issue of including primary outcomes in design and/or maintaining those outcomes are not limited to sports nutrition research and also plague registered clinical trials in which 31% of trials identified a discrepancy between the registered and published primary outcome [23]. Our data suggest a critical need to provide more rigidity in the disclosure of primary outcomes in sports nutrition research. Finally, the prioritizing of study outcomes reinforces the hypotheses proposed by investigators and better focuses their findings and presentation of the study results. 

#### 5.2.3. Change Scores

Change or difference scores can be reported in absolute (i.e., mean) or relative (i.e., percentage) terms. Both have their place yet one can argue that change scores can create a bias towards the group with poorer baseline scores. In this scenario, consideration should be given adjusting data relative to potential confounding issues [24]. With respect to reporting, however, the CONSORT guidelines promote the use of confidence intervals [13]. Researchers who wish to report change scores should first use another method such as ANCOVA to test their hypothesis [25,26]. In using this method, the change score can be used as a dependent variable which is more efficient and has better precision [27]. Of the papers detailing changes scores in our analysis (*n* = 99), 41 (41%) reported SD, 10 (10%) reported SEM, 40 (40%) used CI and eight (8%) presented no variance term. Reporting a change score also presents athletes and coaches with an easily translatable term assuming proper statistical analysis.

## 6. Limitations

The main strength of our paper is that we performed a focused analysis of intervention studies in the field of sports nutrition. This limits our ability to generalize to other sport science studies examining interventions designed to improve performance. Further, one could make a case for the inclusion of other journals that should have been included in our investigation. Though readers should use caution when generalizing our findings to other journals we suggest that the reporting of exercise performance studies and publication in other journals emanate from the same or similar labs. Hence, there is no realistic expectation that such studies will demonstrate better reporting characteristics. The take home point from our analysis, however, is the need for more rigorous reporting standards in sports nutrition.

Another potential limitation of our study is that we did not address studies using magnitude based inference [7]. While we initially considered such an analysis, only eight papers used this form of data reporting. These papers did report SD and confidence intervals (CI, 90%) and were included as fulfilling our primary research question. For more on this topic, we refer readers to published pro and con viewpoints [28,29,30]. One might also criticize our analysis for examining only one year of reporting. Similar to our discussion point above, the studies examined during the year 2016 emanate from a variety of labs and authors that have been reporting findings for decades; hence, there is no realistic expectation of better reporting characteristics in previous years. 

Finally, a current point of contention of data reporting is the use or suggested elimination of *p*-values. This did not fall within the intent of our paper, but all of the papers we analyzed reported *p*-values. Moreover, CONSORT guidelines do not recommend the elimination of *p*-value reporting. This too has been discussed elsewhere [4,7,29,31,32,33]. While we reference CONSORT frequently, it was not our intention to compare reporting in sports nutrition to the complete list of CONSORT recommendations. That said, the CONSORT Statement was developed to improve transparency in parallel-group randomized controlled trials and would certainly bear closer scrutiny within the sports sciences as a whole as these guidelines have been widely adopted across many higher tiered journals [4,34,35,36].

## 7. Conclusions

Intervention trials examining sports nutrition adhere poorly to robust clinical trial standards. We interpret these findings to suggest that although researchers continually seek “state-of-the-art” measurement techniques within their study disciplines, time should also be devoted to improving data reporting. We therefore propose the following. First, from a practical point of view, investigators should devote time to increasing their awareness to more contemporary views on data reporting. Per our primary outcome, there is a clear misunderstanding of reporting variance terms that can be easily rectified by eschewing the SEM in conjunction with intervention trials. 

Secondly, publishers, editors, and reviewers should not only be more aware of such issues, but also be more strict in the publication process via a set of succinct guidelines versus being open ended. Publishers in general can insure such standards by providing more exacting reporting guidelines within the “Instructions to Authors” section of journals. We further suggest that much can be learned from the CONSORT Statement and that publishers and editors alike take a more in-depth look at using the CONSORT to enhance reporting in the exercise sciences as a whole. The time has come for the sports sciences to raise the bar of sports nutrition reporting.

## Figures and Tables

**Table 1:** Reporting characteristics of sports nutrition intervention trials.

Variables	Outcomes	Response (*n*)	Percent
Reporting of Treatment Variance	SD	129	59%
	SEM	79	36%
	CI	11	5%
Effect Size Reporting	None Reported	155	71%
	Reported	63	29%
Outcome Stratification	No	188	86%
	Yes	31	14%
Directed Hypothesis	None Reported	134	61%
	Specific/Directed	57	26%
	Non-Specific	28	13%
Change Score Variance (*n* = 99)	SD	41	19%
	SEM	10	5%
	CI	40	18%
	None Reported	8	4%

Abbreviations: Standard Deviation (SD), Standard Error of the Mean (SEM), Confidence Interval (CI).
